# Supplementary material for: Impact on clinical outcomes of renin-angiotensin system inhibitors against doxorubicin-related toxicity in patients with breast cancer and hypertension: A nationwide cohort study in South Korea
Source: PLoS One. 2023 Nov 20;18(11):e0294649. doi: 10.1371/journal.pone.0294649 (PMC10659172; doi:10.1371/journal.pone.0294649)
Supplement: S1 Table — (DOCX) [file pone.0294649.s001.docx]

S1 Table. Definitions of comorbidities, medications, and clinical outcomes

|  | ICD-10-CM code | Diagnostic definition |
| --- | --- | --- |
| Diagnosis |  |  |
| Breast cancer | C50 |  |
| Exclusion criteria^*^ |  |  |
| Metastasis or malignancy of other sites | C00-C49, C51-C79, C81-97 |  |
| Prior cardiac arrest | I46 |  |
| Myocardial infarction or ischemic heart diseases | I21-I24, I252, I255, I256, I258, I259 |  |
| Heart failure or cardiomyopathy | I27, I50, I130, I132, I110, I42-I43 |  |
| Valvular heart diseases | I34, I35, I38, I39 |  |
| Sick sinus syndrome | I495 |  |
| Arrhythmia | I470, I472-479, I48, I490, I495, I498 |  |
| Severe lung disease | J4402, J441, J4482, J4492, J4503, J4513, J46 |  |
| End-stage renal disease or dialysis | N181-185, Z49 |  |
| Liver cirrhosis or hepatic failure | K72, K74, K702-704, K711 |  |
| Hyperthyroidism | E05 |  |
| Stroke | I60-I64 |  |
| Comorbidities^*^ |  |  |
| Hypertension | I10, I11, I12, I13, I15 | Diagnosis code plus antihypertensive drugs |
| *Without hypertension* |  | Neither diagnosis code for hypertension nor antihypertensive drug use (calcium channel blockers, RAS inhibitor, beta-blockers, thiazide and thiazide-like diuretics) |
| Diabetes mellitus | E10-14 | Diagnosis code plus hypoglycemic agents (metformin, sulfonylurea, meglitinides, thiazolidinedione, dipeptidyl peptidase-4 inhibitor, glucagon-like peptide-1 receptor agonist, alpha- glucosidase inhibitor, sodium-glucose cotransporter-2 inhibitor, insulin) |
| Dyslipidemia | E78 | 1) Diagnosis code plus drugs including fibrate, omega-3 fatty acid, and nicotinic acid  2) Statin user |
| Angina | I20, I250, I251 |  |
| Medications |  |  |
| RAS inhibitors |  | Alacepril, benazepril, captopril, cilazapril, delapril, enalapril, fosinopril, lisinopril, moexipril, ramipril, imidapril, perindopril, quinapril, spirapril, trandolapril, zofenopril, valsartan, losartan, azilsartan, candesartan, telmisartan, eprosartan, fimasartan, irbesartan, olmesartan |
| Antithrombotic agents |  | Aspirin, clopidogrel, cilostazol, ticlopidine, prasugrel, ticagrelor, ticlopidine, triflusal, warfarin, enoxaparin, apixaban, dabigatran, edoxaban, rivaroxaban |
| Statins |  | Atorvastatin, rosuvastatin, simvastatin, fluvastatin, pitavastatin, pravastatin, cerivastatin |
| Outcome |  |  |
| Heart failure | I50, I130, I132, I110, I420 | Diagnostic code plus loop diuretics ≥ 2 (furosemide, torsemide) |

^*^Admission ≥1 or outpatient department ≥2; ICD-10-CM, International Classification of Disease-10th Revision-Clinical Modification; RAS, renin-angiotensin system
