# Supplementary material for: Impact on clinical outcomes of renin-angiotensin system inhibitors against doxorubicin-related toxicity in patients with breast cancer and hypertension: A nationwide cohort study in South Korea
Source: PLoS One. 2023 Nov 20;18(11):e0294649. doi: 10.1371/journal.pone.0294649 (PMC10659172; doi:10.1371/journal.pone.0294649)
Supplement: S2 Table — (DOCX) [file pone.0294649.s002.docx]

S2 Table. Covariates for propensity score matched cohorts

| Characteristics | Non-HT vs. HT (cohort 1) | | | | Non-HT vs. RAS-i (cohort 2) | | | |
| --- | --- | --- | --- | --- | --- | --- | --- | --- |
|  | Non-HT | HT | SMD | | Non-HT | RAS-i | SMD | |
|  |  |  | *before* | *after* |  |  | *before* | *after* |
| Subjects, n | 7,722 | 7,722 | - | - | 4,392 | 1,464 | - | - |
| Age, years | 55 ± 8 | 54 ± 8 | 1.153 | 0.068 | 55 ± 8 | 54 ± 8 | 1.033 | 0.050 |
| Index year, n (%) |  |  | 0.020 | 0.014 |  |  | 0.256 | 0.038 |
| *2008–2010* | 2,185 (28) | 2,187 (28) |  |  | 867 (20) | 311 (21) |  |  |
| *2011–2013* | 3,048 (40) | 3,092 (40) |  |  | 1,730 (39) | 571 (39) |  |  |
| *2014–2015* | 2,489 (32) | 2,443 (32) |  |  | 1,795 (41) | 582 (40) |  |  |
| Diabetes mellitus, n (%) | 785 (10) | 866 (11) | 0.535 | 0.034 | 657 (15) | 247 (17) | 0.649 | 0.052 |
| Dyslipidemia, n (%) | 2,046 (27) | 2,017 (26) | 0.837 | 0.009 | 1,624 (37) | 542 (37) | 0.991 | 0.001 |
| Angina, n (%) | 293 (4) | 322 (4) | 0.357 | 0.019 | 189 (4) | 80 (6) | 0.327 | 0.054 |
| Antithrombotic agents, n (%) | 929 (12) | 1,114 (14) | 0.851 | 0.071 | 672 (15) | 281 (19) | 0.773 | 0.103 |
| Statins, n (%) | 1,955 (25) | 1,923 (25) | 0.806 | 0.010 | 1,557 (36) | 518 (35) | 0.962 | 0.001 |

Non-HT, without hypertension; HT, hypertension; RAS-i, renin-angiotensin system inhibitor; SMD, standardized mean difference
