# Supplementary material for: Impact on clinical outcomes of renin-angiotensin system inhibitors against doxorubicin-related toxicity in patients with breast cancer and hypertension: A nationwide cohort study in South Korea
Source: PLoS One. 2023 Nov 20;18(11):e0294649. doi: 10.1371/journal.pone.0294649 (PMC10659172; doi:10.1371/journal.pone.0294649)
Supplement: S3 Table — (DOCX) [file pone.0294649.s003.docx]

S3 Table. Mean follow-up and differential anticancer therapies between the two groups after propensity score matching

| Covariables | Non-HT vs. HT (cohort 1) | | | | Non-HT vs. RAS-i (cohort 2) | | | |
| --- | --- | --- | --- | --- | --- | --- | --- | --- |
|  | Non-HT | HT | SMD | | Non-HT | RAS-i | SMD | |
|  |  |  | *before* | *after* |  |  | *before* | *after* |
| Mean follow-up, years | 7.62 ± 2.77 | 7.76 ± 2.77 | 0.016 | 0.050 | 7.30 ± 2.59 | 7.46 ± 2.57 | 0.155 | 0.065 |
| Cancer therapy |  |  |  |  |  |  |  |  |
| *High-dose DOX* | 1,477 (19) | 1,448 (19) | 0.049 | 0.010 | 822 (19) | 238 (16) | 0.112 | 0.065 |
| *Concomitant TRA* | 1,850 (24) | 1,795 (23) | 0.052 | 0.017 | 1,054 (24) | 386 (26) | 0.121 | 0.055 |
| *RT in the Lt. chest field* | 300 (4) | 306 (4) | 0.020 | 0.004 | 183 (4) | 70 (5) | 0.051 | 0.030 |

Non-HT, without hypertension; HT, hypertension; RAS-i, renin-angiotensin system inhibitor; Dox, doxorubicin; TRA, trastuzumab; RT, radiotherapy; Lt., left
