# Supplementary material for: Impact on clinical outcomes of renin-angiotensin system inhibitors against doxorubicin-related toxicity in patients with breast cancer and hypertension: A nationwide cohort study in South Korea
Source: PLoS One. 2023 Nov 20;18(11):e0294649. doi: 10.1371/journal.pone.0294649 (PMC10659172; doi:10.1371/journal.pone.0294649)
Supplement: S4 Table — (DOCX) [file pone.0294649.s004.docx]

S4 Table. Sensitivity test: the 1-year primary outcome for each propensity score-matched cohort

| HRs | Non-HT vs. HT (cohort 1) | | Non-HT vs. RAS-i (cohort 2) | |
| --- | --- | --- | --- | --- |
|  | Non-HT | HT | Non-HT | RAS-i |
| Primary outcome = HF + Death | | | | |
| *Crude HR (95% CI)* | 1 (1.00–1.00) | 1.04 (0.83–1.30) | 1 (1.00–1.00) | 0.56 (0.33–0.96) |
| *Adjusted HR (95% CI)* | 1 (1.00–1.00) | 1.04 (0.83–1.30) | 1 (1.00–1.00) | 0.56 (0.33–0.95) |

HR, hazard ratio; CI, confidence interval; Non-HT, without hypertension; HT, hypertension; RAS-i, renin-angiotensin system inhibitor; HF, heart failure
